# Increased ventral anterior insular connectivity to sports betting availability indexes problem gambling

**DOI:** 10.1111/adb.13389

**Published:** 2024-03-22

**Authors:** Damien Brevers, Chris Baeken, Antoine Bechara, Qinghua He, Pierre Maurage, Guillaume Sescousse, Claus Vögele, Joël Billieux

**Affiliations:** ^1^ Louvain for Experimental Psychopathology Research Group (LEP), Psychological Sciences Research Institute UCLouvain Louvain‐la‐Neuve Belgium; ^2^ Department of Behavioural and Cognitive Sciences, Institute for Health and Behaviour University of Luxembourg Esch‐sur‐Alzette Luxembourg; ^3^ Department of Psychiatry UZ Brussel Brussels Belgium; ^4^ Department of Head and Skin, Ghent Experimental Psychiatry (GHEP) Lab Ghent University Hospital, Ghent University Ghent Belgium; ^5^ Department of Electrical Engineering Eindhoven University of Technology Eindhoven The Netherlands; ^6^ Department of Psychology University of Southern California California Los Angeles USA; ^7^ Faculty of Psychology Southwest University Chongqing China; ^8^ Lyon Neuroscience Research Center—INSERM U1028—CNRS UMR5292, PSYR2 Team University of Lyon Lyon France; ^9^ Institute of Psychology University of Lausanne Lausanne Switzerland; ^10^ Centre for Excessive Gambling, Addiction Medicine Lausanne University Hospitals (CHUV) Lausanne Switzerland

**Keywords:** brain connectivity, fmri, insular cortex, problem gambling, reward availability, sports betting

## Abstract

With the advent of digital technologies, online sports betting is spurring a fast‐growing expansion. In this study, we examined how sports betting availability modulates the brain connectivity of frequent sports bettors with [problem bettors (PB)] or without [non‐problem bettors (NPB)] problematic sports betting. We conducted functional connectivity analyses centred on the ventral anterior insular cortex (vAI), a brain region playing a key role in the dynamic interplay between reward‐based processes. We re‐analysed a dataset on sports betting availability undertaken in PB (*n* = 30) and NPB (*n* = 35). Across all participants, we observed that sports betting availability elicited positive vAI coupling with extended clusters of brain activation (encompassing the putamen, cerebellum, occipital, temporal, precentral and central operculum regions) and negative vAI coupling with the orbitofrontal cortex. Between‐group analyses showed increased positive vAI coupling in the PB group, as compared with the NPB group, in the left lateral occipital cortex, extending to the left inferior frontal gyrus, the anterior cingulate gyrus and the right frontal pole. Taken together, these results are in line with the central assumptions of triadic models of addictions, which posit that the insular cortex plays a pivotal role in promoting the drive and motivation to get a reward by ‘hijacking’ goal‐oriented processes toward addiction‐related cues. Taken together, these findings showed that vAI functional connectivity is sensitive not only to gambling availability but also to the status of problematic sport betting.

## Graphical abstract

We conducted functional connectivity analyses centred on the ventral anterior insular cortex (vAI) to examine how sports betting availability modulates the brain connectivity of individuals with or without problematic sports betting. Across all participants, sports betting availability elicited positive vAI coupling with extended clusters of brain activation and negative vAI coupling with the orbitofrontal cortex. Between‐group analyses showed increased positive vAI coupling in problem sports bettors in the inferior frontal gyrus, anterior cingulate gyrus and frontal pole.

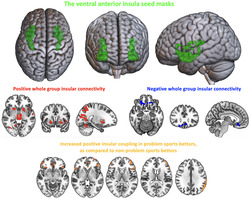

## 1 INTRODUCTION

In recent years, online sports betting has become increasingly popular and widespread among the general population, including in young adults and adolescents.^1^, ^2^ An inherent feature of this type of gambling is that it combines macro (e.g., betting on the outcome of a game) and micro (e.g., in‐play betting) sports outcomes with the prospect of winning money.^3^ Furthermore, with the advent of online technology design features, it is now possible to place a bet on almost any sporting event, at any time and any place.^4^ In this context where sports betting is ubiquitous, merely perceiving an environmental cue related to a sport event (e.g., a game schedule) can act as a powerful incentive to bet.^4^ Accordingly, this increased accessibility to digitalized forms of gambling behaviours is fuelling the level of dysregulated and problematic sports betting behaviours among sports fans.^5^

In a previous functional magnetic resonance imaging (fMRI) study, we employed a cue exposure paradigm where we manipulated gambling availability through a design making sport events available or unavailable for betting.^6^ We showed that brain reactivity to sports betting availability was modulated by the level of problem sports betting status. Specifically, unavailable sport events generally elicited higher activation than available ones among frequent sports bettors with problem gambling (i.e., problem bettors; PB), as compared with frequent sports bettors without problem gambling (i.e., non‐problem bettors; NPB). The cluster of activation encompassed the dorsal striatum, the posterior insula and the parahippocampal gyrus. This brain pattern suggests that PB are more sensitive to the transient inaccessibility of a betting opportunity than NPB.

In the present study, we further examined the brain dynamic of gambling (un)availability using functional connectivity analyses. Specifically, in addition to examining neural activations triggered by task conditions (i.e., available vs. non‐available bets), a complementary approach is to examine how the functional connectivity between brain regions (i.e., psycho‐physiological interaction analyses, PPI^7^, ^8^) is modulated by specific task conditions, and whether the functional coupling between brain areas differs according to the level of problem sports betting (i.e., PB vs. NPB). In other words, PPI analyses allow the identification of functional brain networks, rather than the mere functional brain activity.^9^ Here, we were specifically interested in identifying the brain areas that interact with the ventral anterior insular cortex (vAI) when frequent sports bettors view sports events that are available for betting, as compared with sports events that are not available for betting.

**FIGURE 1:**
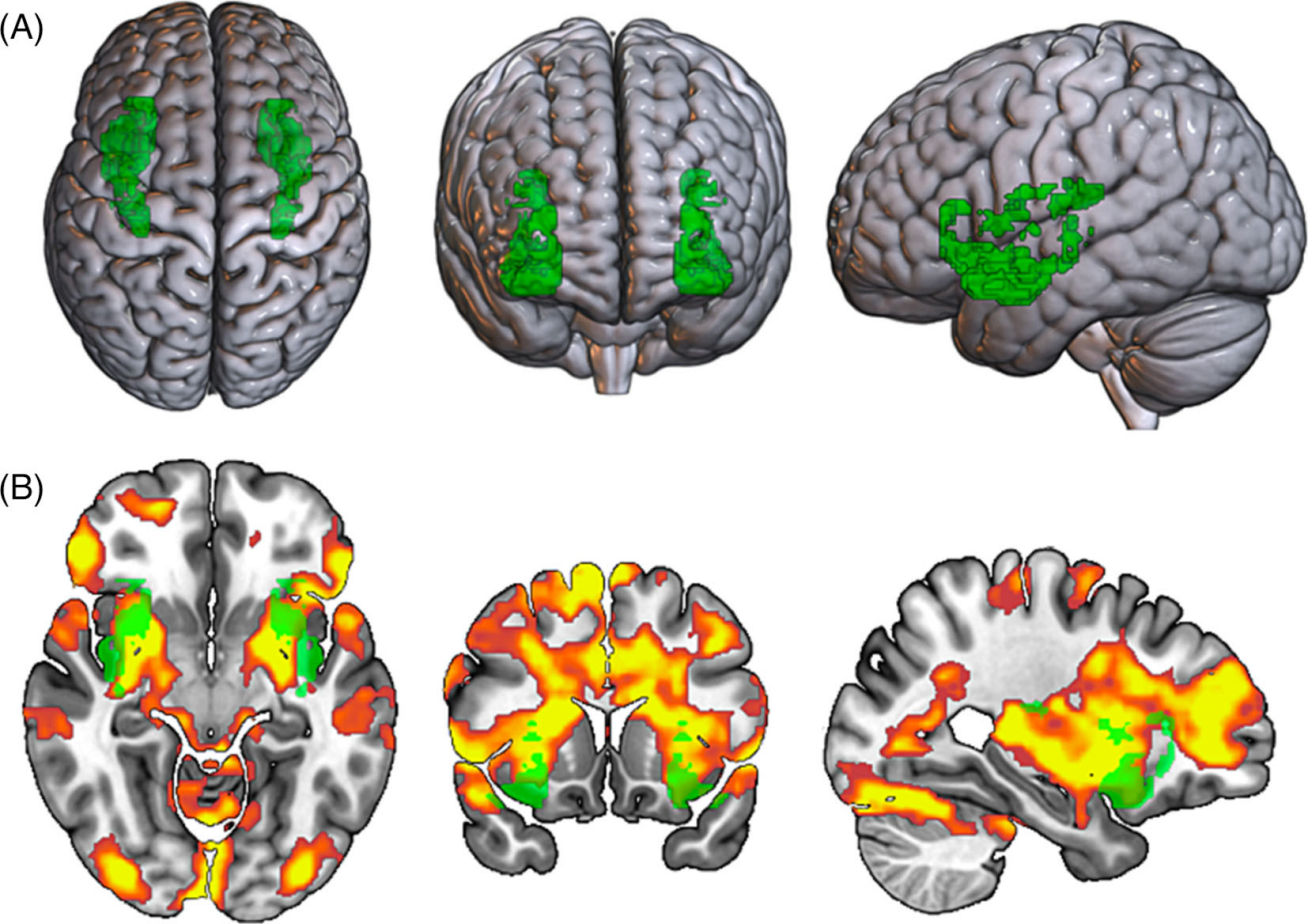
(A) Illustration of the bilateral vAI seed mask obtained from Chang et al.^14^ https://neurovault.org/collections/13/; the bilateral vAI mask is available at https://neurovault.org/collections/KLCKYNIT/); (B) overlap between the vAI seed mask (in green) and pattern of brain activation obtained by Brevers et al.^6^ on the ‘available minus non‐available betting’ contrast.

Indeed, the insular cortex constitutes a ‘gating system’ in the interplay between neurocognitive processes.^10^, ^11^, ^12^, ^13^ Specifically, the insula has been divided into three major subregions that serve distinct functions, with the posterior insula (PI) specializing in the processing of afferent bodily information, the dorsal anterior insula (dAI) in higher‐order executive control cognitive processes and the vAI in emotional and reward‐based processes.^10^, ^11^, ^12^, ^13^, ^14^, ^15^, ^16^, ^17^ Importantly, several functional connectivity studies provided evidence for the hypothesis that the insular cortex is a key hub for identifying interactions among the brain networks involved in the processing of salient‐motivational cues (for a review, see previous works^10^, ^11^). For instance, Zhao et al.^13^ highlighted that during monetary‐based decision‐making, the PI is connected to brain regions involved in sensorimotor motor processes (e.g., supplementary motor area), the dAI is predominately connected with brain regions commonly involved in higher cognition and executive control processes (e.g., dorsolateral prefrontal cortex), and the vAI is preferentially connected to regions from the reward‐brain system, including the ventral striatum and the orbitofrontal cortex (i.e., the fronto‐striatal pathway; e.g., Weber et al.^18^). Hence, the vAI should specifically inform on the pattern of functional connectivity triggered by reward processing. We thus selected the vAI as a seed region for running PPI analyses (see Figure 1A) and posit that this brain region allows for identifying patterns of functional connectivity triggered by the transient availability of a reward, which corresponds here to a sports betting opportunity.

To examine this research question, we conducted vAI‐centred PPI analyses using the dataset of our previously published fMRI on sports betting availability among PB (*n* = 30) and NPB (*n* = 35).^6^ In this previous work, we found extended bilateral vAI activations when contrasting cues available for betting against cues non‐available for betting across the whole groups of participants (*N* = 65; see Figure 1B). Based on this pattern of insular activation, we decided to focus the present PPI analyses on the contrast ‘available betting minus non‐available betting’. Based on the literature on insular‐based PPI, we could expected that, for our whole sample and by contrast with sports cues non‐available for betting, sports cues available for betting would trigger increased positive coupling between the vAI and regions involved in the brain‐reward pathway, including the ventral striatum and the orbitofrontal cortex. However, in our previous study,^6^ we observed higher orbitofrontal and ventral striatal activation in the non‐available condition, rather than the available betting condition. It might thus be possible that a negative coupling between the vAI and these two brain regions is observed. Accordingly, by using a whole brain approach, we expected to observe increased vAI PPI with the orbitofrontal cortex and the ventral striatum during sports betting availability. Based on existing evidence, we expect a positive and negative vAi coupling with these regions (especially with the orbitofrontal cortex).^10^, ^11^, ^12^, ^13^, ^14^, ^15^, ^16^, ^17^ In addition, based on previous fMRI findings obtained with our sports betting availability task, negative PPI could be expected. These positive and negative dynamics of brain connectivity likely reflect the complex nature of the interactions between the insula and the so‐called bottom‐up impulsive and top‐down reflective systems. Specifically, dual‐process models posit that the motivational salience carried by reward‐related cues may (i) sensitize or exacerbate the activity of the reward‐based ‘impulsive’ limbic system and (ii) lower down high‐order cognitive resources of the prefrontal ‘reflective’ system.^19^, ^20^, ^21^, ^22^, ^23^ Nevertheless, reward cue reactivity does not necessarily lead to weaker or hypoactive cognitive control but may instead redirects attention and executive control resources toward reward consumption‐related goals, thereby leading to an increase of activation within prefrontal ‘reflective’ regions.^24^, ^25^, ^26^, ^27^, ^28^ The insula has emerged as a primary neural hub in these dynamic interplays between limbic and prefrontal systems. Specifically, the (anterior) insula integrates autonomic and visceral signals into reward‐motivational functions,^29^, ^30^ which could modulate the balance between the impulsive and reflective neural systems when facing reward‐related cues.^10^, ^11^, ^31^ Accordingly, we have no a priori hypotheses regarding the directionality of the pattern of insular‐based connectivity (i.e., either positive or negative PPI). We combined an exploratory (i.e., directionality of the PPI) and a confirmatory (i.e., the focus on the ventral anterior part of the insular cortex, the expected vAI coupling with the orbitofrontal cortex and the ventral striatum) approaches to examine the patterns of functional connectivity triggered by the transient availability of a reward.

In addition to examining vAI functional connectivity in the context of gambling availability across a whole sample of frequent sports bettors, the second main goal of this study is to examine whether patterns of vAI PPI differ according to the level of problem sports betting (i.e., PB vs. NPB). Triadic models of addiction advance that the insular cortex plays a pivotal role in promoting the drive and motivation to get a reward by sensitization in the brain's mesolimbic and mesocortical dopamine ‘impulsive’ systems and by ‘hijacking’ goal‐oriented ‘reflective’ processes toward addiction‐related cues at the expense of inhibitory control resources.^32^, ^33^, ^34^, ^35^, ^36^, ^37^, ^38^, ^39^, ^40^ Specifically, across repetitions of gambling behaviour, stimuli that signal the accessibility of a gambling‐related reward induce sensitization in the brain's mesolimbic and mesocortical dopamine systems.^35^, ^41^, ^42^, ^43^, ^44^ This level of incentive salience is especially high in individuals who maintain their level of gambling habits despite experiencing gambling‐related harms. This pattern has been demonstrated by neuroimaging studies that highlighted increased functional connectivity in individuals with gambling disorder when exposed to gambling‐related cues (for a review, see the literature^24^, ^45^). For example, Limbrick‐Oldfield and colleagues^46^ observed increased *positive* functional coupling between the insular cortex, and ‘impulsive’ (the striatum) and ‘reflective’ (the superior frontal gyrus) brain regions in problem gamblers, as compared with non‐gambling controls, when these individuals were exposed to gambling‐related cues. We thus expect that, as compared with NPB, PB will exhibit higher positive vAI functional connectivity, and especially with the ventral striatum, when viewing sport cues available for betting. Accordingly, we employed a confirmatory approach (i.e., the focus on the ventral anterior part of the insular cortex, the expected positive vAI coupling with the ventral striatum) to examine the differential patterns of functional connectivity triggered by the transient availability of a reward between NPB and PB.

## 2 METHODS

### 2.1 Participants

The dataset is taken from the 65 football (i.e., soccer) fans that participated in Brevers et al.^6^ study (61 males, mean age 26.04 years, standard deviation [SD] = 5.63, range: 19–51; see supporting information for details on the recruitment procedure; gender and age data are available at https://osf.io/dkrhw). All participants gave written informed consent to the experimental procedure, which was approved by the institutional review boards of University of Luxembourg (ERP 19‐035) and Ghent University (EC/2019/0410). All participants were right‐handed and had normal or corrected‐to‐normal vision. We excluded participants who self‐reported past or present treatment for problem gambling. Participants were advised to avoid drinking alcohol in the 24 h prior to participating in the scanning session. Participants received a fixed amount of 50 euros as a compensation for their participation, plus the money actually won in the sports betting task (up to 20 euros). Participants filled out the Problem Gambling Severity Index (PGSI)^47^ while reflecting on their sports betting behaviours. Based on clinical norms provided by the PGSI,^48^ 35 participants were classified as nonproblem bettors (NPB; PGSI < 3, mean = 0.57, SD = 0.66; range: 0–2), and 30 as moderate to high‐risk gamblers (labelled problem bettors, PB; PGSI ≥ 3, mean = 6.33, SD = 3.21; range: 3–17; PGSI data are available at https://osf.io/dkrhw).

### 2.2 Experimental task and MRI procedure

**FIGURE 2:**
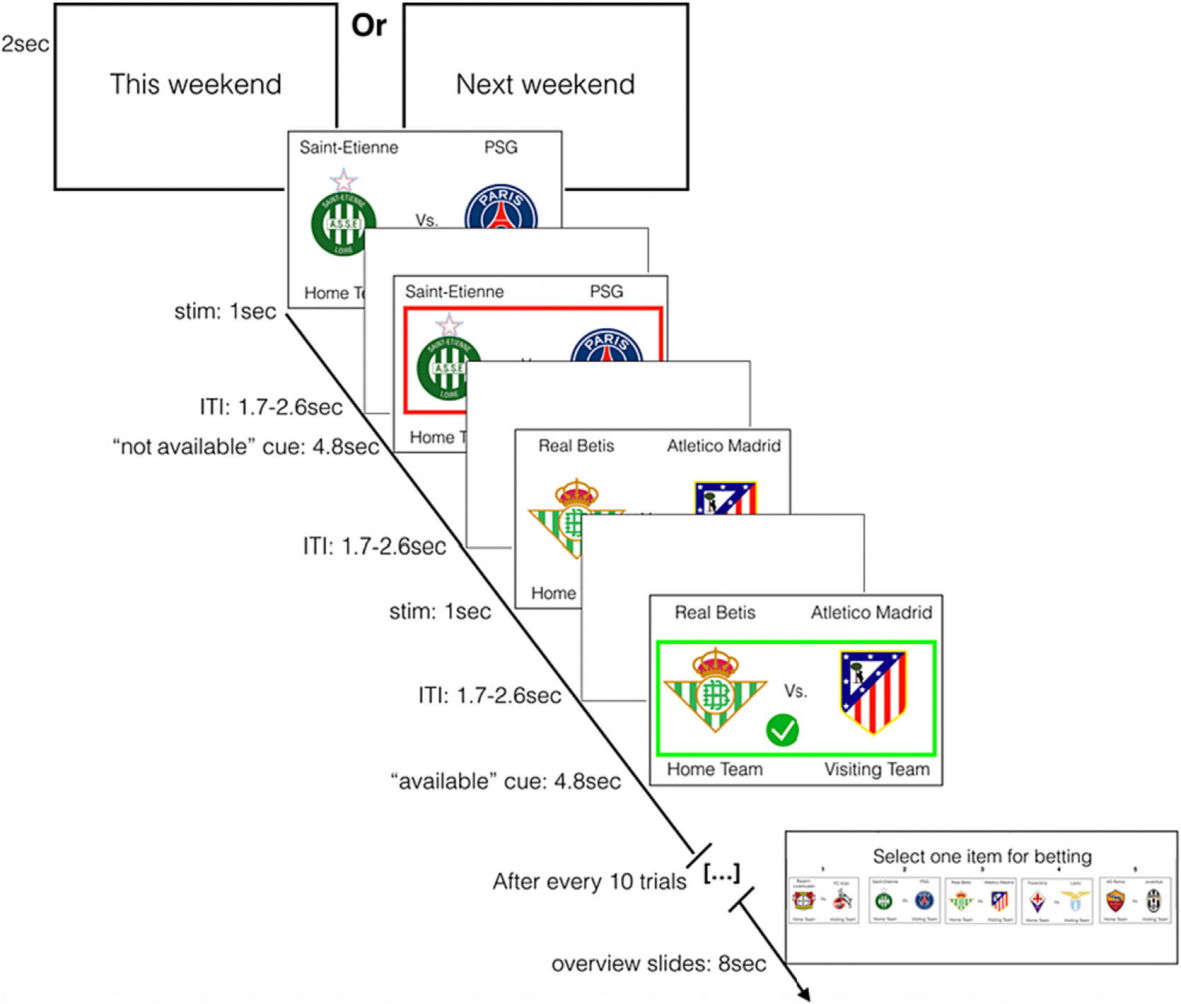
Cue‐exposure task. Examples of sport cues used and of one overview slide. Participants viewed cues representing real sport events that will take place soon and made available or blocked for betting. Participants were instructed to choose, after a run of 10 trials, the team they wanted to bet on. The red frame and the cross signal a trial non‐available for betting. The green frame and the check mark indicates a trial available for betting.

The cue‐exposure task from Brevers et al.^6^ (see Figure 2) depicted football games cues that appeared on a screen (task length ≈ 18 min and 40 s). Prior to the scanning session, participants received task instructions. We asked them to look attentively at each cue and informed them that the task consists of two types of trials, ‘available’ and ‘non‐available’. The games displayed in the ‘available’ condition were available to the participant for betting at the end of a 10‐trial block. The games in the ‘non‐available’ condition were not open for betting; instead, participants were asked to merely observe. See supporting information for a detailed description of the cue‐exposure task and MRI procedure.

### 2.3 Data acquisition and image pre‐processing

These MRI methodological steps correspond to those employed and described in Brevers et al.^6^ This information is fully detailed in supplementary materials.

### 2.4 vAI centred brain connectivity analyses

We performed PPI analyses on the task contrast ‘available minus non‐available betting’ (i.e., the blood‐oxygen‐level‐dependent, BOLD, activity during the 4.8 s onset of ‘available betting’ and ‘non‐available betting’ trials; see Figure 2). To this aim, we modelled the brain imaging data using an event‐related general linear model (GLM) within FSL's improved linear model (FILM) module. We first transformed bilateral vAI seed mask (obtained from Chang et al.^14^; see Figure 1A) into individual space using FLIRT. Next, a time series of significantly activated voxels in the vAI seed mask was extracted for each participant. A first‐level PPI model was then set up using FSL including the following user‐specified regressors: (1) the time course of the seed region; (2) the parametric regressor coding for the task contrasts and (3) the regressor coding the interaction term, that is, the positive and negative multiplications of time course and the task contrast. Single‐subject contrast images for each of these regressors were created.

Each subject's PPI contrast image for the interaction regressor was then entered into a second level random‐effect model for group analysis across all participants (*N* = 65). We also computed a second level random‐effect model for between‐group analysis to compare NPB (*n* = 35) to PB (*n* = 30). All group analyses were performed using FSL FMRIB's local analysis of mixed effects (FLAME 1), with a height threshold of *z* > 3.1 and a cluster probability of *p* < 0.05 (as recommended by Eklund et al.^49^), family‐wise error (FWE) corrected for multiple comparisons across the whole brain.

## 3 RESULTS

### 3.1 Whole group PPI findings

**FIGURE 3:**
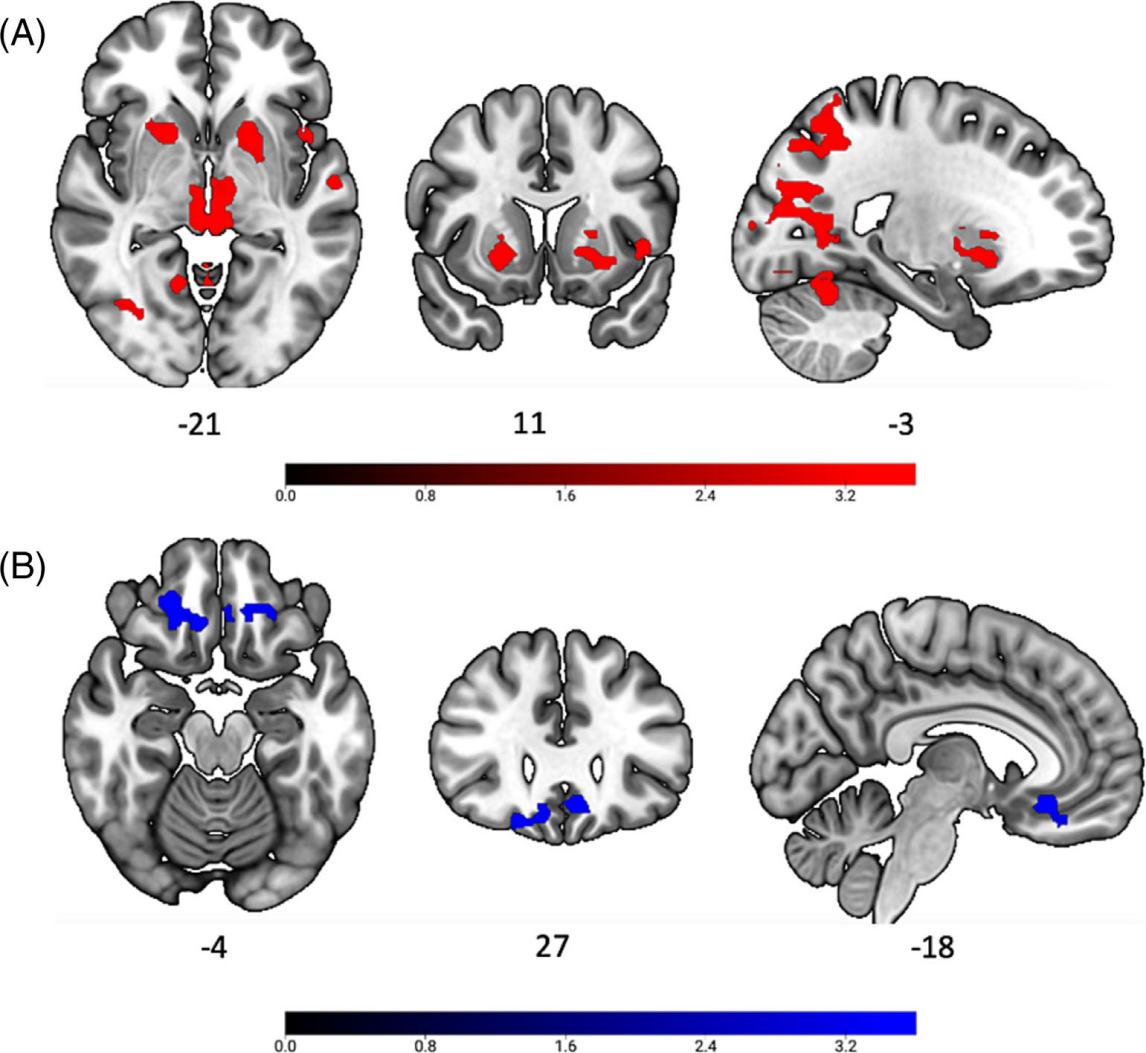
**Whole group PPI.** A, there was extended pattern of positive vAI‐centered PPI (in red); B, negative vAI‐centred PPI with the orbitofrontal cortex (in blue). All images were thresholded using FSL FLAME with a height threshold of *z* > 3.1 and a cluster probability of *p* < 0.05, FWE corrected for multiple comparisons across the whole brain. Left on right.

Across the whole sample (*N* = 65), for the ‘available minus non‐available trials’ contrast, the analyses identified both positive and negative PPI (see Figure 3). We observed a positive PPI (see Figure 3A) between the vAI seed and cluster peak activation in the left lateral occipital cortex (voxel cluster size = 2,764, peak = −20, −62,54; *Z*_max_ = 5.40; voxel cluster size = 1,334, peak = −26, −74,26; *Z*_max_ = 4.76), cerebellum (voxel cluster size = 1711, peak = −8, −60, −22; *Z*_max_ = 4.69), left precentral gyrus (voxel cluster size = 671, peak = −50, −6,24; *Z*_max_ = 5.24; voxel cluster size = 163, peak = 50, −10,36; *Z*_max_ = 3.89), brainstem (voxel cluster size = 575, peak = −4, −28, −4; *Z*_max_ = 5.11), left superior temporal gyrus (voxel cluster size = 475, peak = −52, −20, 4; *Z*_max_ = 4.70), left frontal operculum cortex (voxel cluster size = 270, peak = −44, 10, 0; *Z*_max_ = 6.65), right superior temporal gyrus (voxel cluster size = 232, peak = 56, −16, 2; *Z*_max_ = 4.81) and right putamen (voxel cluster size = 144, peak = 20, 12, −6; *Z*_max_ = 4.41). Negative PPI (see Figure 3B) was observed between the vAI seed and the orbitofrontal cortex (voxel cluster size = 219, peak = 18, 32, −20; *Z*_max_ = 4.53; voxel cluster size = 135, peak = −6, 24, −14; *Z*_max_ = 4.40).

To further determine the directionality of the PPI findings, we undertook additional PPI analyses with the two simple contrasts: ‘available betting (minus implicit baseline)’; ‘non‐available betting (minus implicit baseline)’. We created two region of interest (ROI) masks from the cluster of voxels with significant positive (ROI_PPI_positive) and negative (ROI_PPI_negative) PPI for the ‘available minus non‐available’ contrast, respectively (see **Figure** **S1Ai** in supplementary materials). Using these two masks, we performed two separate ROI analyses (with a height threshold of *z* > 3.1 and a cluster probability of *p* < 0.05) on the ‘available betting’ and the ‘non‐available betting’ contrasts. When using the ROI_PPI_positive mask for the ‘available betting’ contrast, we observed significant positive PPI in all clusters of voxels obtained with the ‘available minus non‐available’ contrast (see **Figure** **S1Aii** in supplementary materials). When undertaken for ‘non‐available betting’ contrast, the ROI_PPI_positive mask resulted in a less extended positive PPI or an absence of significant positive PPI in the clusters of voxels obtained with the ‘available minus non‐available’ contrast (see **Figure** **S1Aiii** in supplementary materials). No significant negative PPI was observed with ROI_PPI_positive mask (for either ‘available betting’ or ‘non‐available betting’). When using the ROI_PPI_negative mask for the ‘available betting’ contrast, we observed significant negative PPI in the orbito‐frontal cortex cluster (see **Figure** **S1Bii** in supplementary materials). When undertaken for ‘non‐available betting’ contrast, no significant negative PPI was observed in the orbito‐frontal cortex. No significant positive PPI was observed with ROI_PPI_negative mask (for either ‘available betting’ or ‘non‐available betting’). These supplementary analyses confirm that the ‘available betting’ condition triggered increased (positive and negative) PPI, as compared with the ‘non‐available betting’ condition.

### 3.2 Between‐group PPI findings

**FIGURE 4:**
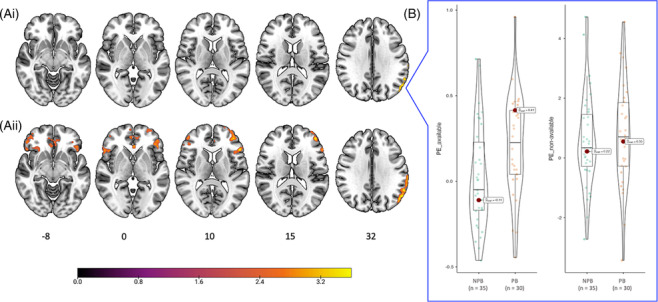
Between‐group PPI differences. (Ai) when using a threshold of *z* > 3.1, a between‐group difference was observed in the left lateral occipital cortex; (Aii) when using a height threshold of *z* > 2.3, between‐group differences extended in the left inferior frontal gyrus, the anterior cingulate gyrus, and the right frontal pole. (B) The red dot of box plots represent mean parameter estimate (PE) in the NPB (*n* = 35) and PB (*n* = 30) groups within the cluster of voxels showing significant activation in the left lateral occipital cortex (obtained with the threshold of *z* > 3.1) for the ‘available minus baseline’ (left panel) and the ‘non‐available minus baseline’ (right panel) contrasts. Left on right.

We observed a between‐group difference in the left lateral occipital cortex (voxel cluster size = 164, peak = −58, −66, 32; *Z*_max_ = 4.02; see Figure 4, Ai). When using a more lenient height threshold of *z* > 2.3 (see Figure 4, Aii), we observed an extension of the between‐group difference in left and right inferior frontal gyri (voxel cluster size = 835, peak = −48, 38, 4; *Z*_max_ = 4.10), anterior cingulate gyrus (voxel cluster size = 562, peak = −2, 32, −4; *Z*_max_ = 3.71) and frontal pole (voxel cluster size = 482, peak = 46, 48, 10; *Z*_max_ = 3.45).

To determine the directionality of the PPI between‐group effect, we created an ROI mask from the cluster of voxels with significant between‐group PPI effect obtained in the left lateral occipital cortex. Using this mask, we performed ROI analyses (with a height threshold of *z* > 3.1 and a cluster probability of *p* < 0.05) by extracting parameter estimates (PE) for each participant and separately for the additional whole brain simple contrasts: ‘positive PPI for available trials’; ‘negative PPI for available trials’; ‘positive PPI for non‐available trials’; and ‘negative PPI for non‐available trials’. We only obtained significant ROI activation for the contrasts ‘positive PPI available’ and ‘positive PPI non‐available’. We then plotted the mean PE in group (NPB and PB) obtained for these two contrasts (see Figure 4B). In addition, post‐hoc independent sample *t*‐tests revealed moderate evidence for between‐group difference on the PE for the contrast ‘positive PPI for available trials’ (Bayes factor 10 = 3.32, Cohen's *d* = 0.62), but no evidence for between‐group difference for the contrast ‘positive PPI for non‐available trials’ (Bayes factor 10 = 0.27, Cohen's *d* = 0.11).

## 4 DISCUSSION

This study examined ventral anterior insular (vAI) functional connectivity patterns in the context of sports betting availability. These PPI patterns were investigated by capitalizing on an existing database from a previous study by Brevers et al.^6^ where a sample of frequent sports bettors with (PB) and without problem gambling (NPB) were exposed to sports cues that were made available or unavailable for betting.

By using a whole brain approach across the whole sample of participants, we observed both positive and negative vAI‐centred PPI when sports bettors were exposed to sports events that were available for betting. The positive pattern of PPI includes extended clusters of brain activation, including the putamen, the cerebellum, occipital, temporal, precentral and central operculum regions. Such extended patterns of PPI signal the multi‐process nature of vAI‐centred brain network that was triggered by sports betting availability. In striking contrast, the negative pattern of vAI PPI only concerns the frontal orbitofrontal cortex. Hence, as expected, we observed vAI coupling with striatal and orbitofrontal regions. The striatal activation was located in the ventral part of right putamen, but not in the nucleus accumbens (i.e., ventral striatum) as predicted. However, like the ventral striatum, the ventral parts of the putamen and caudate, are also largely connected to ventral prefrontal and limbic regions thought to be involved in motivation and emotion, respectively.^50^

These patterns of striatal and orbitofrontal vAI coupling complete well the findings obtained in our previous study through functional brain activity analyses.^6^ Specifically, supplementary PPI analyses undertaken separately for whole brain simple contrasts (i.e., ‘positive PPI for available trials’; ‘negative PPI for available trials’; ‘positive PPI for non‐available trials’; and ‘negative PPI for non‐available trials’) revealed that our observed between‐group PPI effect is driven by an increased positive vAI coupling to available betting in the PB group compared with the NPB group. An intriguing finding from our previous study was the higher activation in the orbitofrontal cortex and in the ventral striatum observed for non‐available betting, as compared with available betting (see also Brevers et al.^25^ for comparable findings). Here, we show that striatal and orbitofrontal regions are also involved in the processing of sports betting availability when coupled with the insula‐based neural network. Furthermore, the positive striatal and negative orbitofrontal PPI patterns further suggest that the insula acts as a key hub for the dynamic interactions between the brain connectivity networks involved in the processing of salient‐motivational cues.^10^, ^11^ Indeed, the observation of both positive and negative PPI not only offers new insights on brain mechanisms underlying humans' reactivity to sports betting cue exposure^6^, ^25^, ^51^ but is also in accordance with brain imaging studies showing that the orbitofrontal cortex and the ventral striatum are involved in the coding of a variety of loss and reward dynamics.^18^, ^52^, ^53^, ^54^, ^55^, ^56^, ^57^, ^58^, ^59^, ^60^, ^61^, ^62^

When examining the effect of problem gambling status, and in line with our expectations, we observed increased positive vAI PPI in the PB group, as compared with the NPB group. Nevertheless, in contrast to our expectations these between‐group PPI, differences were not observed in the ventral striatum, but in left lateral occipital cortex, and extended to left inferior frontal gyrus, anterior cingulate gyrus and right frontal pole when we employed a more lenient threshold. This finding remains important as it suggests that increased vAI connectivity to gambling availability is sensitive to problem gambling status. These results also provide a deeper understanding of the brain dynamics underlying gambling cue activity in problem gambling. In particular, our previous work highlighted that PB are more sensitive to the transient unavailability of a betting opportunity than NPB.^6^ Here, we show that the vAI‐centred functional network is more sensitized to the transient availability of a betting opportunity in PB, as compared with NPB. These complementary patterns likely reflect the complex nature of brain dynamics underlying addictive disorders, which do not exclusively lead to either hypoactive or hyperactive patterns of brain activation or brain connectivity (for a review, see the literature^24^, ^63^). This dynamic is also in line with the main assumption from the triadic models of addictions advance that the insular cortex plays a pivotal role in promoting the drive and motivation to get a reward by ‘hijacking’ goal‐oriented processes toward addiction‐related cues.^35^, ^37^, ^38^, ^64^ This dynamic should lead to increased or decreased insular coupling depending on whether a brain region triggers cognitive resources allowing for the enactment of addiction‐related behaviours.^37^, ^38^, ^45^

A limitation of the present findings is that functional connectivity does not allow to infer causality on the PPI patterns. Nevertheless, the PPI patterns observed in this study allowed to identify seed regions that could be used for running effective connectivity analyses,^31^ allowing to shed light on the causal relation of the observed vAI coupling in our task.^65^ Further studies should also include a control condition that does not require participants to be engaged in sports betting cue exposure (e.g., passive exposure to the sports events cues). This design would enable to further identify the pattern of insula effective and functional connectivity that is triggered by the ‘available betting’ conditions. Another potential limitation of this study is that our sample was mostly constituted of male participants, which hampers generalization of the present results to the population of female football fans. It is also important to extend this research to a sample of individuals at both extreme ends of the spectrum of problem sports betting gambling. Specifically, a central goal of this study was to compare insular‐based PPI between two groups of football fans classified as non‐problem bettors (PGSI <3), or problem bettors (PGSI ≥3) using the clinical norms provided by the PGSI. This recruitment procedure resulted in PGSI scores that were not normally distributed (Chapiro Wilk *p* < 0.001), with only 8% (*n* = 6) of our sample with a PGSC score of ≥8 (i.e., corresponding to moderate level of problem gambling; PGSI data are available at https://osf.io/dkrhw). Hence, future studies should recruit a sample with PGSI scores distribution that matches the proportion of problem sport betting observed in the population of sports bettors^1^, ^66^, ^67^ and ranging continuously at both extreme ends of the spectrum of problem sports betting severity. This would allow the adoption of a dimensional—rather than categorical—approach by examining how the magnitude of the PPI effect is modulated by the degree of problem sports betting severity. Moreover, the brain *Z*‐maps of the present study (available at https://neurovault.org/collections/KLCKYNIT/) can be used as ROI masks by future studies assessing group activation differences in predefined regions of interest in participants with high‐levels of problematic sports betting habits, that is, hard to recruit participants resulting in small and underpowered samples. These studies would allow us to examine whether the pattern of insular‐based connectivity of gambling availability is modulated in the same way in individuals with problem gambling who are active users, compared with individuals who are trying to reduce or stop gambling. Indeed, a challenge for sports fans trying to reduce or stop betting is to watch sports events without betting on them.^68^ In addition, the brain imaging literature has already shown that neural cue reactivity is modulated by the current clinical status of the participant (active user, trying to quit and striving for abstinence). For example, drug‐related cues elicit increased brain activation in individuals who are actively using drugs and not seeking treatment at the time of testing, as compared with treatment‐seeking drug users.^63^, ^69^ Therefore, recruiting sports bettors who differ in their clinical status should allow for further understanding of the role of the insular network in the processing of salient‐motivational cues in problem sports betting behaviours.

In conclusion, the present findings add new insights to the brain imaging literature on problem gambling by identifying the insular‐centered brain circuitry triggered by the availability of sports betting in individuals with different levels of sports betting harm.

## AUTHOR CONTRIBUTION

Damien Brevers, Chris Baeken, Antoine Bechara, Qinghua He, Pierre Maurage, Guillaume Sescousse, Claus Vögele and Joël Billieux participated to the conception and design of the work. Damien Brevers and Chris Baeken were involved in data acquisition. Damien Brevers, Qinghua He and Guillaume Sescousse undertook the data analysis. Damien Brevers, Chris Baeken, Antoine Bechara, Qinghua He, Pierre Maurage, Guillaume Sescousse, Claus Vögele, and Joël Billieux participated to data interpretation and on the writing of the manuscript. All authors approve final version of the manuscript prior its submission.

## CONFLICT OF INTEREST STATEMENT

All authors declare no conflicts of interest relevant to this manuscript.

## Supporting information


**Figure S1.** **(Ai)** region of interest mask (in green) from the cluster of voxels with significant positive PPI for the ‘available minus non‐available’ contrast (ROI_PPI_positive mask); **(Aii)** significant positive PPI for the ‘available betting’ contrast when using the ROI_PPI_positive mask, **(Aiii)** significant positive PPI for the ‘non‐available betting’ contrast when using the ROI_PPI_positive mask; **(Bi)** region of interest mask (in green) from the cluster of voxels with significant negative PPI for the ‘available minus non‐available’ contrast (ROI_PPI_negative mask); **(Bii)** significant negative PPI for the ‘available betting’ contrast when using the ROI_PPI_negative mask. All images were thresholded using FSL FLAME with a height threshold of z > 3.1 and a cluster probability of *p* < .05, FWE corrected for multiple comparisons across the whole brain. Left on Right.

## Data Availability

The experimental task code and stimuli are available on github: https://github.com/dbrevers/sports_betting_study/tree/task/(un)availability_task. The raw data are available on openneuro: doi:10.18112/openneuro.ds002513.v1.0.0.
