# Supplementary material for: Increased ventral anterior insular connectivity to sports betting availability indexes problem gambling
Source: Addict Biol. 2024 Mar 22;29(3):e13389. doi: 10.1111/adb.13389 (PMC11061852; doi:10.1111/adb.13389)
Supplement: Supplementary file 1 — Figure S1. (Ai) region of interest mask (in green) from the cluster of voxels with significant positive PPI for the ‘available minus non‐available’ contrast (ROI_PPI_positive mask); (Aii) significant positive PPI for the ‘available betting’ contrast when using the ROI_PPI_positive mask, (Aiii) significant positive PPI for the ‘non‐available betting’ contrast when using the ROI_PPI_positive mask; (Bi) region of interest mask (in green) from the cluster of voxels with significant negative PPI for the ‘available minus non‐available’ contrast (ROI_PPI_negative mask); (Bii) significant negative PPI for the ‘available betting’ contrast when using the ROI_PPI_negative mask. All images were thresholded using FSL FLAME with a height threshold of z > 3.1 and a cluster probability of p < .05, FWE corrected for multiple comparisons across the whole brain. Left on Right. [file ADB-29-e13389-s001.docx]

***Supplementary materials***

**Participants recruitment**

Participants were recruited via the Internet through advertisements displayed on social media. The ads asked for self-identified football fans (i.e., individuals who like watching European Football) to participate in a neuroimaging study on sports betting. Individuals who were interested were then asked to complete an online survey assessing: (a) the frequency of football game watching, (b) the frequency of football game betting, (c) the knowledge on European Football Leagues, (d), the level of interest for each of the main European Football Leagues. All participants were judged to be physically healthy based on their answers on an MRI screening form, enclosed in the online survey. The pre-screening tool was also used to exclude any participant who reported having used mood stabilizers, antidepressants,

antipsychotics, sleep medications, morphine, cocaine, heroin, or cannabis (regular use) in the past 12 months. The online pre-screening tool is accessible through the following link (in Dutch and French): http:// [www.panlablimesurvey.ugent.be/PAN200/index.php/477436/lang-nl](http://www.panlablimesurvey.ugent.be/PAN200/index.php/477436/lang-nl). The screening procedure allowed to identify participants who watch football frequently (6-points Likert scale; all participant scores either “5—frequently” or “6—very frequently”) and maintain a high level of knowledge and interest in European Football Leagues. Regarding the frequency of football betting, participants ranged from nonfrequent to highly frequent betters. Specifically, on a 5-points Likert scale, 28 participants reported to bet “3—sometimes” on football, 25 participants reported to bet “4—frequently” on football, and 12 participants reported to bet “5—very frequently” on football.

**Experimental task and MRI procedure**

The cue-exposure depicted football games from a European league (i.e., English Premier League, German Bundesliga, Italian Calcio, French Ligue 1, Spanish Liga, Portuguese Primeira Liga, Dutch Eredivisie, and Belgian ProLeague) that was about to occur either the same weekend of the scanning session (referred here as “weekend 1”) or the next one (“weekend 2”). We chose games occurring on two consecutive weekends to be able to select up to 100 games from the main European leagues. Therefore, all participants were scanned on a Saturday (9 am–6 pm, 7–10 participants per day), and different games were displayed on each scanning session. Seven Saturday sessions were conducted in total (between April 2019 and September 2019).

Participants were first presented with those game cues for 1 s (showing the logos of the two teams playing), and after a jittered delay (blank screen, range: 1.7–2.6 s), an “available” cue (green frame and check mark) or “nonavailable” cue (red frame and cross signal) was presented for 4.8 s (see **Figure 1** in the main manuscript). Each block consisted of 10 pseudorandomized trials (5 “available” and 5 “nonavailable” trials; order pseudo-randomized with Python's random generation module). Each scanning block terminated with an overview slide (8 s), displaying the five available matches presented during the block (see **Figure 1**). During this phase, participants orally reported the number of one game and the team that they wanted to bet on (e.g., “One, Chelsea”) via intercom to the experimenter. Participants were informed that they would receive the betting money once the sport event that he bet on had occurred (2 euros for a win; 1 euro for a draw, 0 euro for a loss). Thus, they could win from 0 euro (10 losing bets) up to 20 euros (10 winning bets). Finally, participants were informed that the task consisted of 10 blocks (100 trials in total), and that there were five blocks displaying games occurring during the scanning session weekend (“weekend 1”; indicated by “this weekend” appearing for 2 s at the beginning of the block; see Figure 1), and five blocks displaying games occurring the next weekend (“weekend 2”; indicated by “next weekend” appearing for 2 s at the beginning of the block). The “weekend 1” and “weekend 2” blocks were presented in alternating order (5 s white screen between blocks).

Several specific strategies were employed for preventing participants motion during the MRI session: (a) participants were instructed to relax and to remain still during the entire scanning session; (b) during the task instructions, the experimenters trained the participants on how to express their choice orally (during the overview slide) with a reduced level of mouth movements; and (c) within the head coil, participants head was secured and stabilized with supportive cushions. In addition, participants wore headphones (to facilitate communication with the experimenter), which further prevented (head) motion within the head coil during the MRI session. We also put a rest leg on the scanner table for participants' comfort.

Directly after the scanning session, participants were asked to complete rating scales. For each of the 50 games in the “available” and “nonavailable” conditions, participants were requested to indicate (a) which team they think would win the game (by circling the team; there was not the option to choose a draw), and (b) how confident they were about their prediction (1 = not at all, 2 = very little, 3 = somewhat, 4 = to a great extent). Then, participants completed the Problem Gambling Severity Index (PGSI; Ferris and Wynne, 2011), and indicated their bank account number for receiving the bonus payment from their bets (conducted after weekend 2).

**Data acquisition**

Cues presentation was implemented using Python 2.7.16 and Pygame 1.9.3 on an IBM compatible PC. fMRI imaging was conducted with a 3T Siemens MAGNETOM Prisma scanner at the GIfMI Center, UZ Gent, Gent University. Functional scanning used a z-shim gradient echo EPI sequence with PACE (prospective acquisition correction). This sequence is designed to reduce signal loss in the prefrontal and orbitofrontal areas. The PACE option can help to reduce the impact of head motion during data acquisition. The parameters were: TR = 1720 ms; TE = 27 ms; flip angle = 66 degrees. Fifty-two 2.5 mm axial slices were used to cover the whole cerebral cortex and most of the cerebellum without gap. The slices were tilted approximately 30 degrees clockwise along the AC-PC plane to improve the signal-to-noise ratio. A 176-slice MPRAGE structural sequence was also acquired (1 mm slice thickness; TI = 900 ms; TR = 2,250 ms; TE = 4.18 ms; flip angle 9 degrees). Prior to the EPI sequence, standard Siemens magnetic field maps were collected with the same slice prescription as the functional scans using a multi-echo gradient echo acquisition (effective EPI echo spacing = 0.52 ms, EPI TE = 27 ms, % signal loss threshold = 10). These field maps were used for correction of geometric distortions in the EPI data caused by magnetic field inhomogeneity.

**Image pre-processing**

Image preprocessing was carried out using the fMRI Expert Analysis Tool (version 6.00, part of the FSL package, FMRIB software library, version 5.0.9, www.fmrib.ox.ac.uk/fsl). The first three sets of each participant's functional data were discarded to allow the MR signal to reach a steady state. Functional data for each participant were motion-corrected using rigid-body registration, implemented in FMRIB Software Library (FSL)'s linear registration tool, MCFLIRT (Jenkinson et al., 2002). All participants (N = 65) demonstrated less than 1.0 mm of either absolute or relative motion. Hence, head motion did not lead to exclude any participant. After motion correction and temporal highpass filtering, each time series for geometric distortions caused by magnetic field inhomogeneity was corrected using field maps (Jenkinson, 2003, 2004). Data were spatially smoothed using a 5-mm full-width-half-maximum (FWHM) Gaussian kernel. The data were filtered in the temporal domain using a nonlinear high pass filter with a 90 s cut-off (estimated using FSL's FMRI Export Analysis Tool, FEAT). A two-step registration procedure was used where EPI images were first co-registered to the MPRAGE structural image, and warped to standard (MNI) space, using FLIRT (Jenkinson et al., 2002; Jenkinson and Smith, 2001). Registration of MPRAGE structural image to MNI standard space was then further refined using FNIRT nonlinear registration (Anderson et al., 2007a, 2007b). Statistical analyses were performed in the native image space, with the **
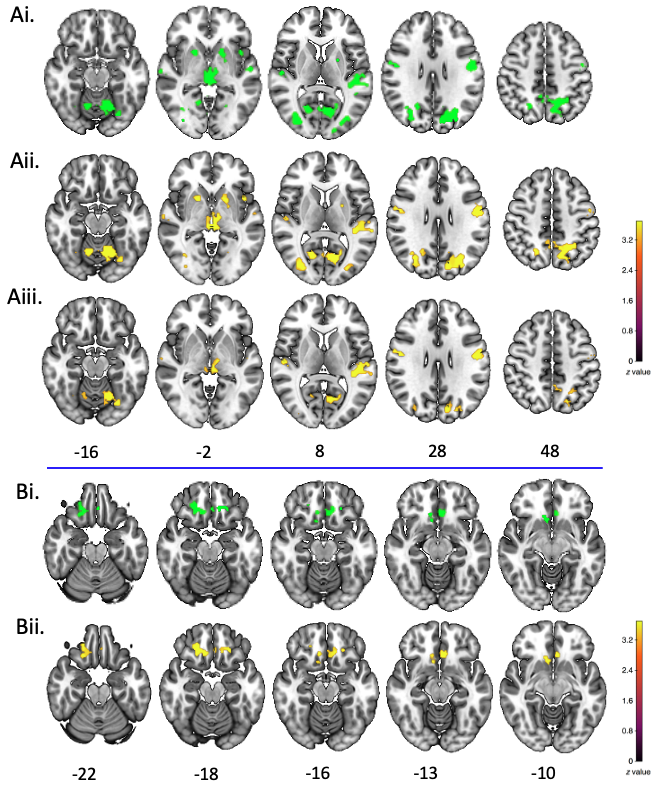
**statistical maps normalized to the standard space prior to higher-level analysis*.*

**Figure S1. (Ai)** region of interest mask (in green) from the cluster of voxels with significant positive PPI for the “available minus non-available” contrast (ROI_PPI_positive mask); **(Aii)** significant positive PPI for the “available betting” contrast when using the ROI_PPI_positive mask, **(Aiii)** significant positive PPI for the “non-available betting” contrast when using the ROI_PPI_positive mask; **(Bi)** region of interest mask (in green) from the cluster of voxels with significant negative PPI for the “available minus non-available” contrast (ROI_PPI_negative mask); **(Bii)** significant negative PPI for the “available betting” contrast when using the ROI_PPI_negative mask. All images were thresholded using FSL FLAME with a height threshold of z > 3.1 and a cluster probability of *p* < .05, FWE corrected for multiple comparisons across the whole brain. Left on Right.

**References**

Ferris J, Wynne H. (2011). *The Canadian Problem Gambling Index: Final report*. Ottawa, ON: Canadian Centre on Substance Abuse.

Jenkinson M, Bannister P, Brady M, Smith S. (2002). Improved optimization for the robust and accurate linear registration and motion correction of brain images. *Neuroimage, 17(*2):825-841.

Jenkinson M. (2003): A fast, automated, n-dimensional phase unwrapping algorithm. *Magnetic Resonance in Medicine, 49*:193-197.

Jenkinson M. (2004). *Improving the registration of B0-disorted EPI images using calculated cost function weights.* Paper presented at Tenth International Conference on Functional Mapping of the Human Brain, Budapest, Hungary.

Jenkinson M, Smith S. (2001). A global optimisation method for robust affine registration of brain images. *Medical Image Analysis, 5*(2):143-156.

Andersson JLR, Jenkinson M, Smith S. (2007a) *Non-linear optimisation (FMRIB Technical Report TR07JA1)*. Oxford, UK: FMRIB Analysis Group Retrieved from [www.fmrib.ox.ac.uk/datasets/techrep.](http://www.fmrib.ox.ac.uk/datasets/techrep.)

Anderson JLR, Jenkinson M, Smith S. (2007b). *Non-linear registration, aka spatial normalisation (FMRIB Technical Report TR07JA2).* Oxford, UK: FMRIB Analysis Group. Retrieved from. [www.fmrib.ox.ac.uk/datasets/techrep](http://www.fmrib.ox.ac.uk/datasets/techrep).
